# Correlations reveal the hierarchical organization of biological networks with latent variables

**DOI:** 10.1038/s42003-024-06342-y

**Published:** 2024-06-03

**Authors:** Stefan Häusler

**Affiliations:** Faculty of Biology and Bernstein Center for Computational Neuroscience, Ludwig-Maximilians-Universität München, Munich, Germany

**Keywords:** Neuroscience, Computational biology and bioinformatics

## Abstract

Deciphering the functional organization of large biological networks is a major challenge for current mathematical methods. A common approach is to decompose networks into largely independent functional modules, but inferring these modules and their organization from network activity is difficult, given the uncertainties and incompleteness of measurements. Typically, some parts of the overall functional organization, such as intermediate processing steps, are latent. We show that the hidden structure can be determined from the statistical moments of observable network components alone, as long as the functional relevance of the network components lies in their mean values and the mean of each latent variable maps onto a scaled expectation of a binary variable. Whether the function of biological networks permits a hierarchical modularization can be falsified by a correlation-based statistical test that we derive. We apply the test to gene regulatory networks, dendrites of pyramidal neurons, and networks of spiking neurons.

## Abstract (web summary)

A method to infer the modular organization of biological networks based on incomplete information. The method is based solely on observable network data and can be used to yield reconstructions of networks obtained from different data sources, such as cellular, genetic and brain measurements.

## Introduction

**Fig. 1:**
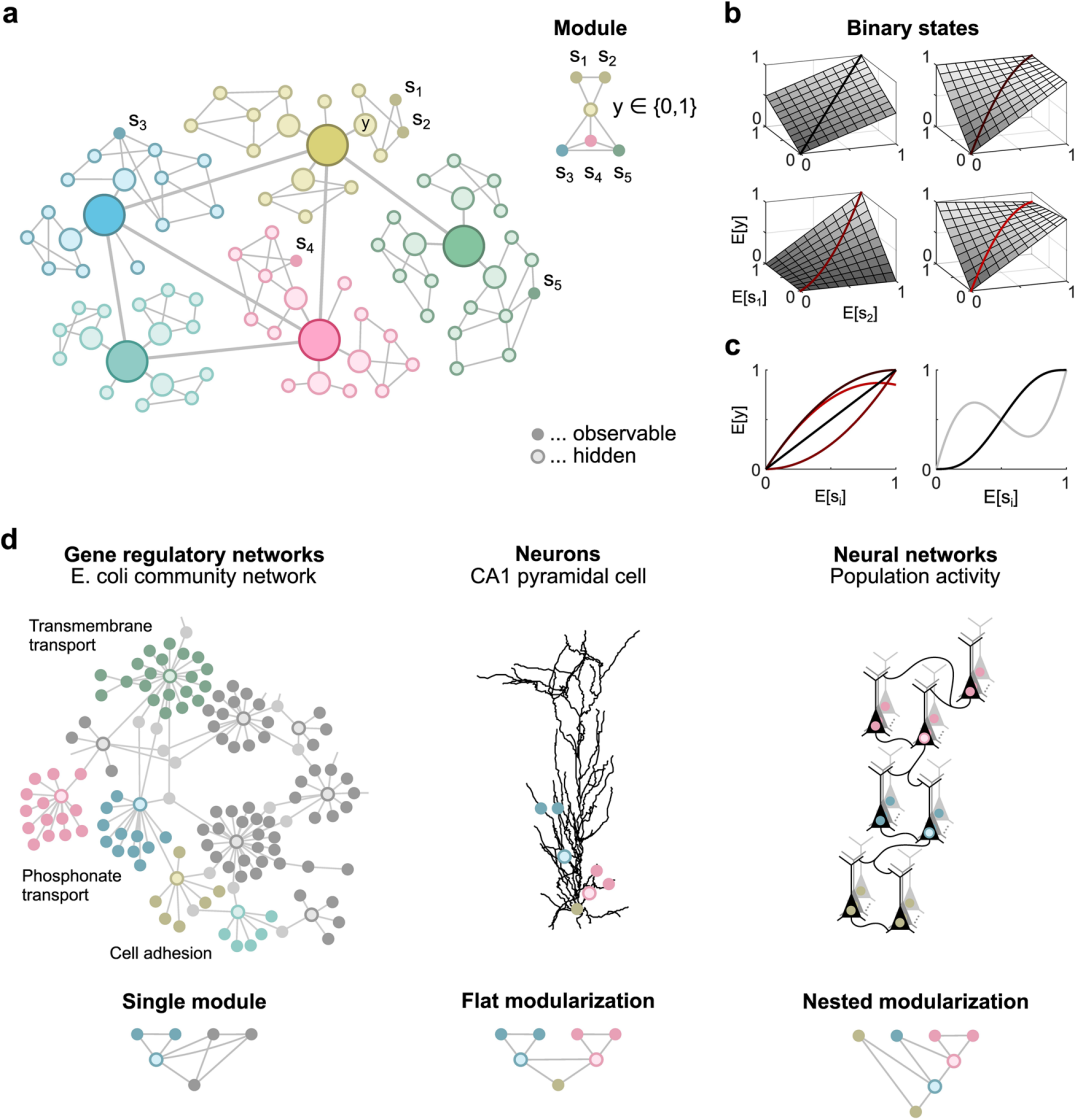
Functional modules in undirected graphical models. **a** Undirected graphical model representing dependencies between network components (left). Right: Functional module consisting of the observable components *s*_1_ and *s*_2_, which are independent of all other observable components given the interface variable *y*. **b** Examples of interface rate functions for the functional module shown in (**a**). **c** Left: Three of the interface rate functions shown in panel **b** are nonlinear, as illustrated for *E*[*s*_1_] = *E*[*s*_2_]. Right: Examples of nonlinear interface rate functions of three (black line) and five (gray line) arguments, illustrated for identical arguments *E*[*s*_*i*_]. **d** Three scenarios used for statistical testing.

Modern recording techniques in neuroscience and cell biology are generating datasets of rapidly increasing dimensionality, posing a major challenge to current analytical methods for deciphering the function of the underlying biological systems^1,2^. A promising approach in graph theory^3–5^ is to decompose and organize complex networks^6^ into largely autonomous functional modules^7^, as found at all levels of biological organization^3,8^. Various heuristic algorithms have been proposed to detect functional modularity, many of them based on hierarchical clustering^9^, but a more rigorous analysis requires exact probabilistic inference^10^. Following this approach, a functional module can be conveniently formalized as a subnetwork that communicates or interacts with the rest of the network only through a particular variable, which we call interface variable. This interface variable may represent, for example, the firing rate of a population of sensory neurons that encodes all information about stimuli relevant to downstream areas. If the value of this interface variable is known, the internal and external components of a module are conditionally independent (Fig. 1a).

However, probabilistic inference of functional modules in large biological networks is challenging. It is often not possible to record from the entire network, and interface variables may be inaccessible. Moreover, these variables can be abstract quantities such as sensory, associative, motor, or cognitive information encoded in the activity of cell populations^11^. And even if the interface variables are recorded, their identification for large networks is computationally intractable for combinatorial reasons. Here, we bypass these problems and investigate whether it is possible to infer functional modularizations from the distribution of observable network components alone, without information about the organization and values of interface variables.

Remarkably, an arbitrary scalar interface variable does not impose any experimentally testable conditions for continuous network states. This is easy to see, as each of the finitely many samples in a dataset can always be mapped to different values of a scalar variable, thus allowing any functional modularization. To avoid this trivial solution, we constrain the interface variables and focus on the simplest case of binary variables.

We show that the functional organization of networks with latent binary interface variables can be inferred from the statistical moments of observable network components alone, and derive a statistical test for hierarchical modularizations. Importantly, this test can also be applied to refute functional modularizations of networks consisting of continuous scalar interface variables if the following two conditions are met.

First, only the mean values of the continuous scalar interface variables and observable components are relevant for the function of the network and thus for its modularization. The actual distribution of the network states conditioned on these mean values is arbitrary as long as it is consistent with the modularization. For many stochastic biological systems, this condition is assumed to be satisfied, e.g., in molecular biology by the rate of gene transcription^12^ and in neuroscience by the instantaneous firing rate of neurons^13^. Second, the mean of a variable downstream of an interface variable depends only linearly on the mean of that interface variable, where downstream refers to any sampling scheme (Fig. 1b). The specific shape of this linear function may depend on other variables, allowing for distributed nonlinear computing (Fig. 1c). In particular for modularizations where a subnetwork depends on the interface variables of several disjoint functional modules, this assumption is satisfied for arbitrary continuous interface variables as long as each of the interface variables contributes only linearly to the mean of each subnetwork component.

Although these assumptions limit the applicability of the method, it is relevant for a number of biological networks. Both assumptions are met by probabilistic Boolean networks, where uncertainties about binary network states are encoded by mean values. Moreover, these assumptions are reasonable when network components are well connected such that a single input has only a small, approximately linear effect on the overall nonlinear activity of a component. Here, we show that the statistical test for modularization is applicable to three biological networks at different spatial scales, and evaluate key hypotheses about their underlying functional organization (Fig. 1d).

## Results

### Functional modules

We describe observable network components by random vectors **s** = (*s*_1_, …, *s*_*d*_) in ${{\mathbb{R}}}^{d}$ and functional modules by sets *S*_*n*_ for *n* = 1, 2,… that contain the indices of all observable components within a module. Associated with each functional module *S*_*n*_ is a potentially hidden binary interface variable *y*_*n*_ that separates its internal components from all other components such that all internal components indexed by *S*_*n*_ are conditionally independent of all other components given *y*_*n*_ (Fig. 1a). A modularization consists of several functional modules and is described by a set ${{{{{{{\mathcal{M}}}}}}}}=\{{S}_{1},{S}_{2},\ldots \}$.

The key question is how to infer functional modularizations from samples of **s** without information about the underlying interface variables **y**, which prevents direct testing of the corresponding conditional independencies. We assume that the observable states are bounded, so that all their moments are finite and uniquely determine the probability distribution of **s**. We first show that functional modules are reflected in pairwise correlations between network components.

### Pairwise correlations indicate direct dependencies

Consider a large neural network where the spike counts of five neurons are observed within some time interval and described by the random components *s*_1_, …, *s*_5_. Furthermore, assume that the first two neurons are part of the same neuronal population such that their expected spike counts, i.e., *E*[*s*_1_] and *E*[*s*_2_], are proportional to an unknown population firing rate *r*, and only this rate drives the rest of the network. As the two components *s*_1_ and *s*_2_ are independent of all other observable components given *r*, they form a functional module. The dependencies between the five observable components are shown in the graphical model in Fig. 1a.

We analyze pairwise correlations of an equivalent network where *r* is replaced by a binary interface variable *y* whose expectation, *E*[*y*], is proportional to *r*. By assumption, the expected components *s*_1_ and *s*_2_ are linear in the population rate *r*, and, thus, all pairwise correlations between the observable components remain unchanged. As the graphical model remains the same, the dependencies between components are also equivalent. Therefore, we can analyze the equivalent network to infer the dependencies in the original network. There are no restrictions on the dependence of the population rate *r* on the observable components outside of the functional module.

**Fig. 2:**
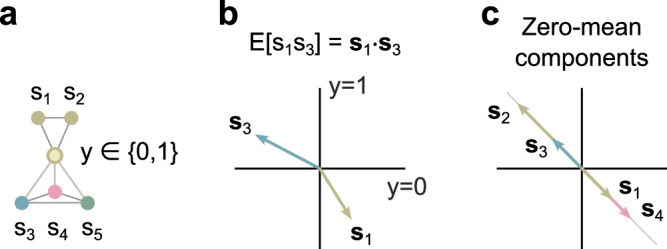
Vector representation of pairwise correlations. **a** Components *s*_1_ and *s*_3_ of the undirected graphical model are conditionally independent given the interface variable *y*. **b** Their pairwise correlation, *E*[*s*_1_*s*_3_], corresponds to the scalar product of the vectors **s**_1_ and **s**_3_. **c** All vector pairs associated with zero-mean network components are either parallel or antiparallel.

The effect of the functional module with binary interface variables on the properties of pairwise correlations can be visualized by a 2D vector representation (Fig. 2). According to the law of total expectation and the conditional independence statement of the module, the correlation of a component within the module, e.g., *s*_1_, and a component outside of the module, e.g., *s*_3_, can be written as a scalar product *E*[*s*_1_*s*_3_] = **s**_1_ ⋅ **s**_3_ of vectors

1 $${{{{{{{{\bf{s}}}}}}}}}_{i}=\left(\begin{array}{c}E[{s}_{i}| y=0]\,\,{{\mbox{P}}}\,{(y = 0)}^{1/2}\\ E[{s}_{i}| y=1]\,\,{{\mbox{P}}}\,{(y = 1)}^{1/2}\end{array}\right)$$

for *i* = 1, 2, …,5. Here, *E*[*s*_*i*_∣*y* = 0] and P(*y* = 0) denote the conditional expectation of *s*_*i*_ given *y* = 0 and the probability of *y* = 0, respectively.

In general, the vector components are unknown because the interface variable *y* is unknown. Yet, if all components are normalized to zero mean *E*[*s*_*i*_] = 0, all pairs of vectors are either parallel or antiparallel (Fig. 2 right). Then, the ratio of **s**_3_ ⋅ **s**_*i*_ and **s**_4_ ⋅ **s**_*i*_ is independent of **s**_*i*_ for *i* = 1, 2 and has an absolute value equal to the ratio ∣**s**_3_∣/∣**s**_4_∣. Therefore, observable components with non-zero pairwise correlations, a functional module *S* = {1, 2} and a binary interface variable *y* exist only if

2 $$\frac{E[{s}_{1}{s}_{3}]}{E[{s}_{1}{s}_{4}]}=\frac{{{{{{{{{\bf{s}}}}}}}}}_{1}\cdot {{{{{{{{\bf{s}}}}}}}}}_{3}}{{{{{{{{{\bf{s}}}}}}}}}_{1}\cdot {{{{{{{{\bf{s}}}}}}}}}_{4}}=\frac{{{{{{{{{\bf{s}}}}}}}}}_{2}\cdot {{{{{{{{\bf{s}}}}}}}}}_{3}}{{{{{{{{{\bf{s}}}}}}}}}_{2}\cdot {{{{{{{{\bf{s}}}}}}}}}_{4}}=\frac{E[{s}_{2}\, {s}_{3}]}{E[{s}_{2}\, {s}_{4}]}.$$

This condition can be tested to reject indirect dependencies between the components *s*_1_ and *s*_2_ and the components *s*_3_ and *s*_4_ via a rate function *r*. Based on estimators of pairwise correlations and estimators of their covariance, we derive an asymptotic test for direct dependencies that can be applied even when pairwise correlations are zero (see Methods).

### Inferring direct interactions in gene regulatory networks

High-throughput technologies, such as RNA sequencing and microarrays, capture transcriptomes under a variety of experimental conditions to infer transcriptional gene regulation. Probabilistic Boolean networks have been successfully applied to infer the underlying gene regulatory networks^14,15^, where expression values of transcription factors (TFs) and target genes (TGs) form nodes (or components), and direct interactions between TFs and TGs form links. We therefore apply the proposed method to this inference task. In order to test in a competitive environment, we retroactively participate in the DREAM5 Challenge^16^, a comprehensive evaluation of 35 network inference methods on various datasets with established gold standards. Here, the interface variables of functional modules are not hidden, but correspond to recorded expression values of TFs, allowing a comparison with inference methods that rely on this information.

The reconstructed networks are compared to experimentally established gold standards for two datasets, *Escherichia coli* and an in-silico benchmark. The submission format of the DREAM5 Challenge is a ranked list of predicted regulatory interactions. Because TF–TF interactions are not organized hierarchically, we restrict the reconstruction to TF–TG interactions, which represent more than 94% of the gold standard. The performance is evaluated using the area under the precision-recall curve (AUPR), the receiver operating characteristic curve and an overall score that summarizes the performance across networks. For a fair comparison with previous results, we evaluate all performance measures against the full gold standard.

To apply the test, we start with a ranked list of direct TF–TG interactions, ordered by the absolute value of their Pearson correlation coefficient, and investigate whether some of these direct interactions can be explained by indirect dependencies through other TFs. More specifically, we investigate all subnetworks consisting of two TFs and two TGs and test for a functional module *S* containing both TFs. Based on the test, the rank of each TF–TG interaction is re-evaluated in such a way that evidence against a functional module, i.e., against an indirect interaction, shifts the rank towards more likely interactions, and reduced evidence shifts the rank in the opposite direction (see Methods). The test is only applied if a subnetwork is sufficiently connected such that at least three of the four putative TF–TG interactions are in the set of the most likely interactions. The size of this set is the only free parameter of the method.

**Fig. 3:**
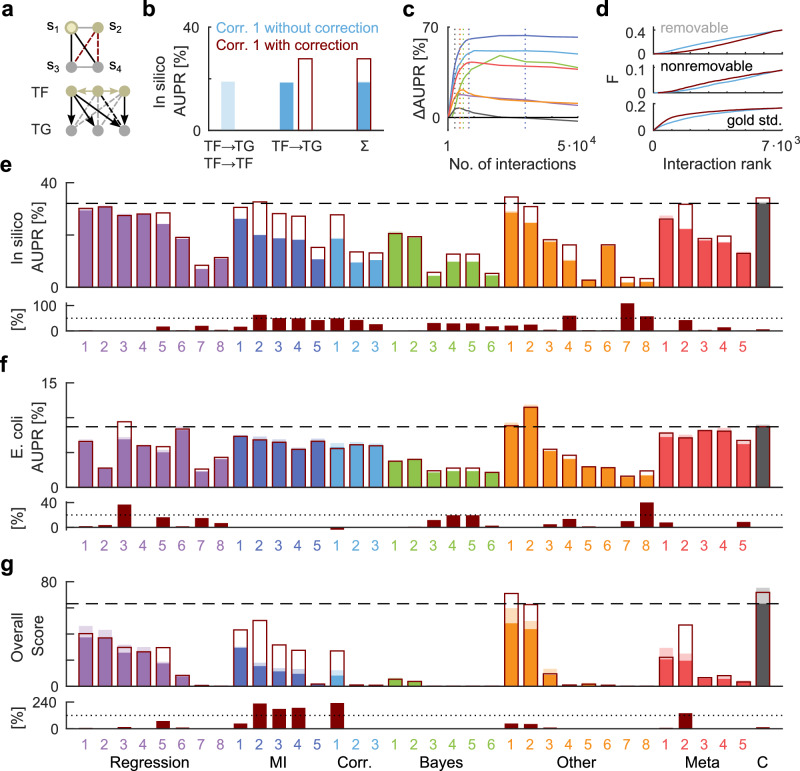
Inference of direct interactions in gene regulatory networks. **a** Undirected graphical model of a subnetwork consisting of four genes (top). Black edges represent putative direct interactions, which are elements of the set of most likely interactions. Red edges represent putative indirect interactions to be tested. Bottom: Example of a directed graphical model representing causal interactions between genes (arrows). Dashed lines indicate removable (gray) and nonremovable (black) indirect interactions. **b** Performance of the uncorrected method based on Pearson correlation coefficients (Corr. 1) for all interactions (light blue) or only for TF-TG interactions (blue) of the in-silico benchmark. The correction based on the test improves the performance by 50% (dark red rectangle). **c** Performance as a function of the size of the set of most likely interactions for the network inference methods Regression 5, MI 2, Corr. 1, Bayes 4, Other 1, Meta 2 and the community network (color code as in **e**). Dotted lines indicate the selected sizes of the sets, determined on a disjoint holdout set. **d** Cumulative distributions of removable, nonremovable and gold standard interactions as a function of the rank in the list of most likely interactions for the corrected (blue line) and uncorrected (dark red line) inference method Corr. 1. **e** Performance of all 36 inference methods of the DREAM5 challenge before (colored bars) and after their correction based on the test (dark red rectangles) for the in-silico benchmark (top). The dashed line indicates the performance of the community network (C). Bottom: Relative performance improvement in %. The dotted line indicates an improvement of 50%. **f** Same as (**e**), but for the *Escherichia coli* dataset. **g** Overall score summarizing the performance across networks and performance measures.

For each subnetwork (Fig. 3a), we denote the expression levels of the two TFs as *s*_1_ and *s*_2_, the expression levels of the two TGs as *s*_3_ and *s*_4_, and use their correlations *E*[*s*_*k*_*s*_*l*_] to test for the functional module *S* = {1, 2}, where *k* and *l* index the observable components inside and outside of the functional module, respectively. Only if the condition in Eq. (2) holds, TF–TG interactions in this subnetwork can be mediated by a single transcription factor with an expression level represented by the probability of a binary variable. We derive a statistical test for this condition, which can also be applied to zero-correlated expression levels (see Methods). Under certain assumptions, the method is asymptotically correct in the sense that the most likely inferred interactions are true TF–TG interactions. There are no constraints on the interface rate functions as long as the interdependence of the co-regulated TGs *s*_3_ and *s*_4_ is linear.

Figure 3b shows the performance (AUPR) of the uncorrected reconstruction of the in-silico network based on all TF-TF and TF-TG interactions ordered by the absolute value of their Pearson correlation coefficients. Omitting all TF-TF interactions from the reconstruction results in about the same performance, while correcting according to the test improves the AUPR by 50%. The test doesn’t require fine-tuning of its only free parameter, the number of most likely interactions that determine whether a subnetwork is sufficiently connected, which is optimized on a holdout set (Fig. 3c).

The effect of the correction can be analyzed in terms of the rates of true negatives and false positives (type I errors). If a TG is regulated by several interdependent TFs, the test might fail to refute a false direct TF–TG interaction because the corresponding functional module has not one but several interface variables. If this is the case according to the gold standard, we call indirect TF-TG interactions nonremovable (Fig. 3a), which account for less than 30% of all TF-TG dependencies. Figure 3d shows that the improvement in performance is due to a majority of removable indirect interactions, whose rank distribution is correctly shifted towards less likely interactions. In contrast, the rank distribution of nonremovable indirect interactions is shifted towards more likely interactions, introducing more likely false positives (type I errors). Overall, the rank distribution of the gold standard is shifted toward more likely interactions.

As the correction only requires a ranked list of predicted regulatory interactions, we apply it to each of the inference methods of the DREAM5 Challenge for the in-silico (Fig. 3e) and *E. coli* microarray data (Fig. 3f). In general, the correction improves most of the 36 inference methods, suggesting that it takes advantage of otherwise unexploited information. In particular, the correction improves the overall score of the community network, which is about the same as that of the single corrected inference method Genie3^17^, denoted as Other 1. However, our aim is not to develop a single best inference method for gene regulatory networks, which will probably be a combination of different inference methods. Rather, we show that this inference method is generally suitable for reconstructing gene regulatory networks and propose it for datasets with missing or unknown regulatory TFs.

### Moment ratios indicate functional modules

To allow the construction of an efficient statistical test, we consider only hierarchically organized modularizations that are either flat or nested. We call a modularization ${{{{{{{\mathcal{M}}}}}}}}$ flat if all functional modules contained in ${{{{{{{\mathcal{M}}}}}}}}$ are disjoint (Fig. 1d). And we call a modularization ${{{{{{{\mathcal{M}}}}}}}}$ nested if all functional modules contained in ${{{{{{{\mathcal{M}}}}}}}}$ are either disjoint or a subset of another functional module in ${{{{{{{\mathcal{M}}}}}}}}$ (Fig. 1d). To clearly distinguish between flat and non-flat nested modularizations, we single out one component, denoted as *s*_ref_, that is not part of any functional module. In the following, *s*_ref_ refers to *s*_*d*_.

We show that moments of **s** uniquely determine whether the observable states form a particular modularization or not (see Methods). Let *P*_*n*_ for *n* = 1, 2, … denote an infinite sequence of all monomials in the observable components within a given functional module, e.g., for the module in Fig. 1a, the sequence *P*_1_ = 1, *P*_2_ = *s*_1_, *P*_3_ = *s*_1_*s*_2_, …. Moreover, let *Q*_*m*_ for *m* = 1, 2, … denote the corresponding sequence of all monomials in the observable components outside of the functional module, e.g., for the module in Fig. 1a, the sequence *Q*_1_ = 1, *Q*_2_ = *s*_3_, *Q*_3_ = *s*_3_*s*_4_, *Q*_4_ = *s*_3_*s*_5_, ….

**Fig. 4:**
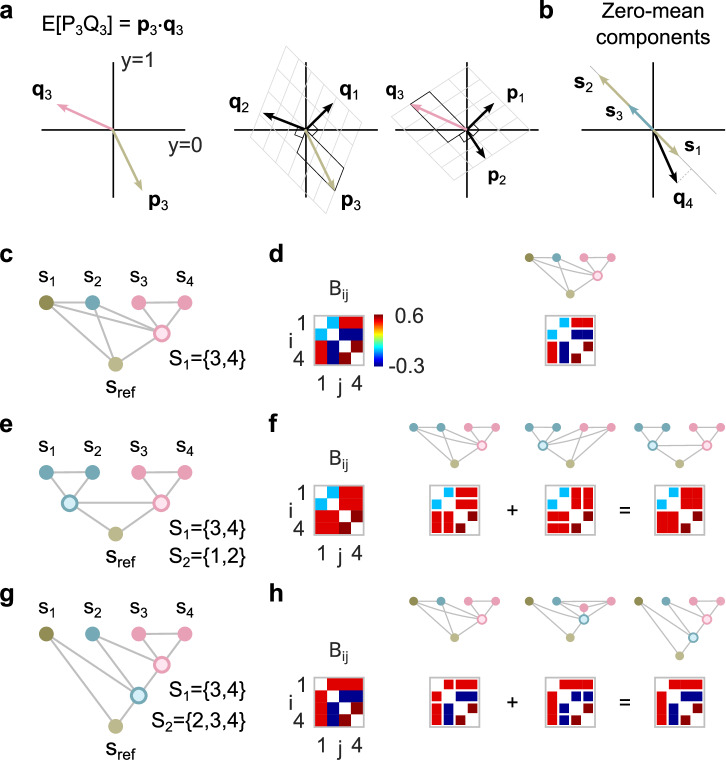
Moment ratios indicate functional modularizations. **a** Left: The moment *E*[*P*_3_*Q*_3_] corresponds to the scalar product of the associated vectors **p**_3_ and **q**_3_. Middle: Vector **p**_3_ expressed in the skewed coordinate system with axes perpendicular to **q**_1_ and **q**_2_. Right: Vector **q**_3_ expressed in the skewed coordinate system with axes perpendicular to **p**_1_ and **p**_2_. **b** All vector pairs associated with zero-mean network components are either parallel or antiparallel. The vector **q**_4_ is associated with a mixed moment of order greater than one and can point in any direction. **c** Single functional module. **d** Example of a moment ratio matrix for the modularization shown in (**c**) (left). Right: Matrix elements with the same value due to the single functional module are shown as connected. **e** Flat modularization. **f** Example of a moment ratio matrix for the modularization shown in panel **e** (left). Right: Each of the two modules implies that different elements of the moment ratio matrix have the same value. The conditions for the moment ratio matrix resulting from the overall modularization are obtained by combining the conditions for the individual modules. **g** Nested modularization. **h** The conditions for the moment ratio matrix resulting from the nested modularization shown in (**g**) are obtained by combining the conditions for the individual modules.

As in the case of pairwise correlations, the moment *E*[*P*_*n*_*Q*_*m*_] is equal to the scalar product **p**_*n*_⋅**q**_*m*_ of the two vectors **p**_*n*_ and **q**_*m*_ defined analogous to Eq. (1). If all observable states have zero mean, all pairs of vectors **s**_*k*_ are either parallel or antiparallel (Fig. 4b), where *k* indexes all observable components inside of the module. Hence, ratios of scalar products of different **q**_*m*_ and the same **s**_*k*_ have equal values for all *k*. For a single functional module *S*, we use the monomials *s*_*k*_ for *P*_*k*_, where *k* indexes all observable components inside of the module. In addition, we use *s*_ref_ for *Q*_ref_ and the monomials *s*_*l*_*s*_ref_ for *Q*_*l*_, where *l* indexes all observable components outside of the module. To enable efficient testing, we introduce a matrix **B** with elements

3 $${B}_{kl}=\frac{{{{{{{{{\bf{p}}}}}}}}}_{k}\cdot {{{{{{{{\bf{q}}}}}}}}}_{l}}{{{{{{{{{\bf{p}}}}}}}}}_{k}\cdot {{{{{{{{\bf{q}}}}}}}}}_{{{{{{{{\rm{ref}}}}}}}}}}\cdot \frac{1}{E[{s}_{l}{s}_{{{{{{{{\rm{ref}}}}}}}}}]}=\frac{E[{s}_{k}{s}_{l}{s}_{{{{{{{{\rm{ref}}}}}}}}}]}{E[{s}_{k}{s}_{{{{{{{{\rm{ref}}}}}}}}}]E[{s}_{l}{s}_{{{{{{{{\rm{ref}}}}}}}}}]}$$

for 1 ≤ *k* < *d* and 1 ≤ *l* < *d*, where we divide each element by *E*[*s*_*l*_*s*_ref_] to obtain a symmetric matrix. Observable components **s** with (finite) moments as above, a functional module *S* and a binary interface variable exist if and only if for each *l* indexing an observable component outside of the functional module *S*, the moment ratios *B*_*kl*_ have the same value for all *k* in *S* (Fig. 4c, d).

Moreover, for flat and nested modularizations consisting of several functional modules, the combined conditions can be derived directly from the conditions of the individual functional modules (Fig. 4e–h). Observable components **s** with moments analogous to those above, a nested (flat) modularization ${{{{{{{\mathcal{M}}}}}}}}$ and a binary interface variable exist only if (if and only if) the corresponding combined conditions are met.

Based on estimators $\hat{{{{{{{{\bf{B}}}}}}}}}$ of the moment ratio matrix **B** and estimators of the covariance matrix of the elements of $\hat{{{{{{{{\bf{B}}}}}}}}}$, we derive an asymptotic test for the conditions in Eq. (3) that can be applied even if some moment ratios do not exist (see Methods). Furthermore, we consolidate numerically that this test is not only asymptotically correct but holds for sufficiently many samples such that the moment estimates are approximately joint normal. For the investigated datasets, a few hundred samples turn out to be sufficient to fulfill these requirements. A more general test using arbitrary moments is given in the Methods.

As a typical use case, the test is applied to multiple candidate modularizations and needs to be corrected for multiple testing (see Methods). Note that the method does not require parameter optimization, only the calculation of the test statistic for each candidate modularization. However, the number of potential single functional modules already grows exponentially with the number of observable components, which can lead to combinatorial problems. We expect that current conventional computing resources can handle up to 24 observable components, resulting in approximately 10^7^ candidate modules. To overcome the combinatorial explosion for flat modularizations, we propose to first identify all single functional modules and then test for all of their combinations. For nested modularizations, we suggest starting with an educated guess of a detailed nested modularization consisting of *n* functional modules and testing for all 2^*n*^ potential nested modularizations obtained by combining these functional modules.

### Inferring modularizations in neural networks: proof of principle

**Fig. 5:**
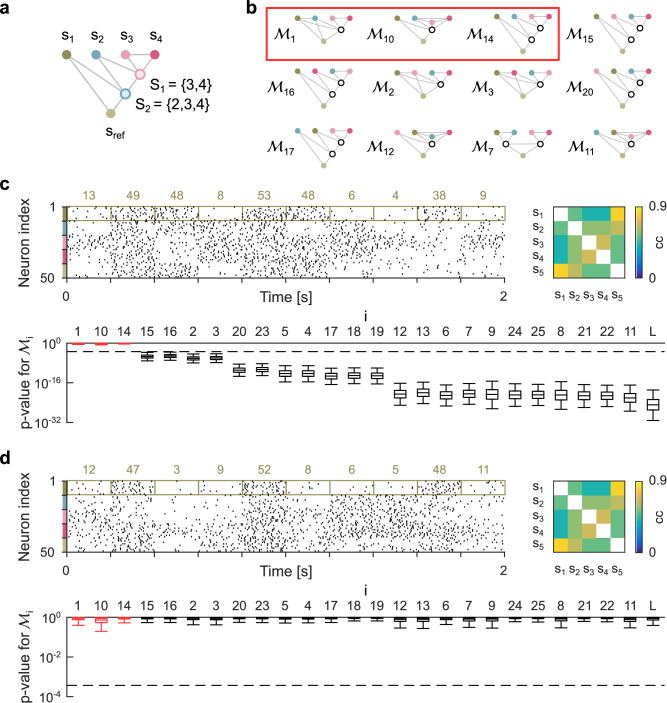
Inference of functional modules in neural networks. **a** Nested modularization consisting of five populations of neurons. **b** 12 out of 26 potential modularizations. Modularizations consistent with the modularization shown in panel **a** are marked with a red rectangle. **c** Spiking activity of all neurons and correlation matrix of observable components (top). Observable states are spike counts within populations and time intervals. Colored numbers represent samples of the observable component *s*_1_. Bottom: Boxplot showing the distribution of *p* values for testing each of the 26 modularizations. Boxes represent the first through third quartiles, and whiskers indicate the 2.5 and 97.5 percentiles. **d** Same as (**c**), but for a linear modularization with the same correlation matrix. Dashed lines represent overall significance levels of 0.01.

As a case study illustrating the power of the approach, we infer the hierarchical organization of a neural network from simultaneously recorded spiking activity. It is a classical hypothesis in neuroscience that neurons communicate information only through their instantaneous firing rates, typically characterized by the spike count within a certain time window or population^13,18,19^. Accordingly, we simulate the spiking activity of five populations of 10 neurons each with dependencies as shown in the undirected graphical model in Fig. 5a. The corresponding interface rate functions are shown in Fig. 1b (see Methods) and can be implemented by a probabilistic Boolean network.

The task is to infer all modularizations that are consistent with the data (Fig. 5b), given recorded spiking activity over 15 min. For simplicity, we follow a classical population coding approach and define the observable network components as the total number of spikes of all neurons recorded within a population during consecutive 200 ms time intervals (Fig. 5c). Likewise, the interface variables are spike counts for latent populations. The corresponding moment ratio matrix $\hat{{{{{{{{\bf{B}}}}}}}}}$ (Eq. (3)) is shown in Fig. 4h, which is consistent with the three modularizations ${{{{{{{{\mathcal{M}}}}}}}}}_{1}$, ${{{{{{{{\mathcal{M}}}}}}}}}_{10}$ and ${{{{{{{{\mathcal{M}}}}}}}}}_{14}$. In contrast, all other modularizations, including a purely linear model ${{{{{{{{\mathcal{M}}}}}}}}}_{{{{{{{{\rm{L}}}}}}}}}$, are rejected by the test at an overall significance level of 0.01 (Fig. 5c).

The test relies on a small number of correlations between observable components to efficiently investigate potential nonlinear dependencies with *s*_ref_. For this approach, pairwise correlations alone are not sufficient to refute any nonlinear modularization. There always exists a random vector with the same pairwise correlations as the observable states that allows any modularization (Fig. 5d).

### Inferring functional modules in dendrites of pyramidal neurons

Pyramidal neurons exhibit complex morphologies and spatially modulated distributions of ion channels^20,21^ that generate regenerative events, such as Na^+^ or NMDA spikes, localized to specific branches or subtrees^22^. Previous work has suggested that these branches act as independent functional modules^23–27^, whose responses to local synaptic inputs are linearly summed at the soma. Simulation studies have confirmed that the resulting flat modularization is indeed an accurate description for computations on firing rates^28,29^, where the input and the response are encoded by the rate of synaptic inputs and somatic action potentials, respectively.

However, these studies required complete information about synaptic inputs and were limited to paired branch stimulation. It is still unclear whether the functional modularization breaks down in scenarios in which a large number of branches are stimulated at the same time. For pulse stimulation applied to pairs of branches in the dendritic tuft, it has been shown that cross-talk between the branches prevents precise functional modules^29^. Here, we investigate the formation of functional modules in proximal and oblique apical dendrites when the pyramidal neuron is excited by strong input to all its terminal branches. In particular, we apply the proposed method not only to infer single functional modules, but the total overall modularization of the proximal apical dendrites.

**Fig. 6:**
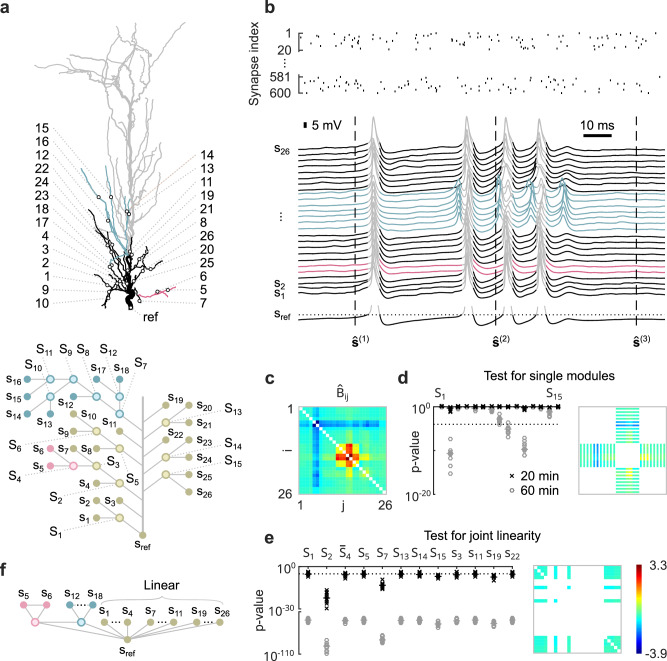
Inference of functional modules in dendrites of pyramidal neurons. **a** Model of the apical dendrites of a CA1 pyramidal neuron (top). The investigated subtree is shown in color and black. Black circles indicate recording locations. Bottom: Undirected graphical model of the largest possible modularization consistent with the morphology of the investigated subtree. **b** Synaptic input to terminal branches (top). Bottom: Observable states are membrane potentials recorded at the corresponding locations shown in panel **a**, sampled every 50 ms. Spiking activity at the soma (gray traces) is excluded from the analysis. **c** Estimated moment ratio matrix. **d** *p* values for testing each of the 15 functional modules shown in panel **a** on a log scale (left). Right: Elements of the moment ratio matrix expected to have the same value according to the single module *S*_7_, represented as contiguous blocks. **e** *p* values for testing whether individual functional modules or dendritic branches originating from the trunk participate in a large linear somatic module (on log scales). All tests are repeated ten times on independent datasets obtained from 20 and 60 min recordings. Dotted lines represent overall significance levels of 0.01. Horizontal bars indicate medians. Right: Elements of the moment ratio matrix expected to have the same value according to a linear module consisting of *S*_1_, *S*_14_, *S*_15_, *s*_3_, *s*_11_, *s*_22_ and ${\bar{S}}_{4}=\{{s}_{4},{s}_{7}\}$. **f** Approximate flat modularization of the proximal apical and oblique dendrites.

More specifically, we simulate a detailed multi-compartment model of a CA1 pyramidal neuron^24^ and stimulate excitatory synapses at terminal branches at a constant rate. The network components correspond to subthreshold membrane potentials at 26 locations in the proximal and oblique apical dendrites, recorded at 50 ms intervals over 20 or 60 min (Fig. 6a, b) to investigate the statistical power of the test for different sample sizes. The interface variables are non-binary, latent and correspond to membrane potentials within functionally independent dendritic compartments downstream of the recording sites that are linearly summed at the soma. As the somatic module is linear and we are testing for flat modularizations, there are no constraints on the interface rate functions.

The estimated moment ratio matrix $\hat{{{{{{{{\bf{B}}}}}}}}}$ (Eq. (3)), obtained from 60 min recordings, shows three nonlinear functional modules, i.e., *S*_4_, *S*_7_ and *S*_11_ (Fig. 6c). The test rejects only three individual functional modules (Fig. 6d) at an overall significance level of 0.01. The first, *S*_2_, is rejected because its two most proximal observable components, *s*_4_ and *s*_7_, are part of a large linear somatic module, while its two distal observable components, *s*_5_ and *s*_6_, form the nonlinear module *S*_4_. The other two rejected modules, *S*_9_ and *S*_11_, are part of the large nonlinear module *S*_7_.

In addition, we investigate which of the functional modules are purely linear, as these can be integrated into a large somatic module. For a purely linear module *S*, the square submatrix of **B** indexed by *S* has identical off-diagonal elements. Then, any functional modularization within *S* is possible, reflecting the commutative property of addition, as multiplication can be excluded for somatic integration. For each functional module, we test whether it is part of a larger linear module consisting of 11 observable components. The constraint ensures the same degrees of freedom for all tests. For 20 min recordings, only modules *S*_2_ and *S*_7_ are not part of the large linear somatic module, in contrast to the proximal part of module *S*_2_ labeled ${\bar{S}}_{4}$ (Fig. 6e).

The resulting flat functional modularization of the proximal apical dendrite consists of a linear somatic module and the two nonlinear modules *S*_4_ and *S*_7_ (Fig. 6f). However, the large linear somatic module is only approximate and rejected for large enough sample sizes.

## Discussion

Unraveling the functional organization of large biological networks is challenged by incomplete information and combinatorial problems. We present an asymptotic test for hierarchical functional organization of network components based on observable correlations alone, which requires no information about latent network components.

The method presented here differs significantly from previous approaches to inferring functional structure in large networks because it is not based on optimization. In neuroscience, network connectivity and hidden variables are traditionally inferred from neuronal activity based on principles such as maximum a posteriori estimation, Bayesian inference, or information theory^2,30,31^. However, such optimization paradigms require regularization in the form of prior information or otherwise prefer more complex structures due to overfitting. Moreover, there is no indication of whether unexplained network activity is due to noise or an inadequate (hierarchical) structure of the latent variables.

In contrast, constraint-based approaches, which first test for conditional independence in the data and then find appropriate network structures, provide intuitive results such as *p* values, but cannot be applied to incomplete data^10^. The method presented here combines the advantages of constraint-based approaches and probabilistic models by providing a statistical test for partially latent network structures. In particular, the method tests not only necessary conditions, potentially refuting any false nested modularization, but also sufficient conditions for flat modularizations of observable network components with the same correlations as used for the test.

In molecular biology, probabilistic Boolean network models have been successfully applied to infer gene regulatory networks^32–34^, but these methods aim at a complete network reconstruction including all logical relationships between genes (logic gates). In contrast, the statistical test presented here does not estimate model parameters. For flat modularizations, only a minimal set of sufficient and necessary conditions is considered, in the sense that omitting a single correlation estimate renders the test inconclusive. We therefore believe it is particularly well suited for small sample sizes, or equivalently, for inferring large functional organizations from datasets of a given size. In particular, the method is useful as a first step in the analysis process to gain an initial understanding of the functional organization of a network.

We test the method against a benchmark for reconstructing gene regulatory networks and show that it outperforms previously superior community-based methods when used as a correction to the single inference method Genie3^17^. In addition, we apply the method to a detailed model of a pyramidal neuron and show that its proximal apical dendrites form multiple functional modules in response to distributed and strong driving synaptic input.

Previous work^29^ analyzed the response of the same pyramidal neuron model to synaptic stimulation of pairs of dendritic branches, allowing a comparison of the functional modularization for the two input types. In this study, only three of the proximal branches are analyzed, but not the branch with the observable components *s*_12_ to *s*_18_ (see Fig. 5a). These branches show a similar, but not identical, flat modularization with a single nonlinear functional module containing the components *s*_5_ to *s*_7_. The larger functional module for paired-branch stimulation may be due to the strong local synaptic input required to activate the neuron, whereas weaker distributed stimulation results in a smaller functional module.

The functional modularization of the pyramidal neuron model can be identified without any information about the synaptic inputs or the electrophysiological properties of the neuron. This makes the method suitable for conventional two-photon microscopy with voltage indicators, which measures subthreshold membrane potentials at unspecific locations defined by the intersection of the neuron and the imaging plane.

The test is based on correlations between network components, which are usually reflected in correlated moment ratios. The stronger these correlations are, the lower is the statistical power of the test. For moderately correlated network components, as in the neural network inference example, a recording duration of about 15 min is sufficient to achieve adequate statistical power. However, the highly correlated network components of the pyramidal neuron model require recording durations of 60 min. In particular, for synaptic integration in the tuft dendrites, the statistical power is too low to refute any functional module for the chosen recording durations.

Probabilistic Boolean networks with univariate interface variables can not capture the complex dependencies between components of many biological neural networks. However, they may be suitable for modeling sensory systems if neuronal populations implement optimal coding schemes for information processing on short time scales^35,36^. Then, the optimal neuronal response functions are binary and intermediate rates reflect states of uncertainty. In particular, binary response functions have been shown to be reasonable approximations for various sensory domains^37^.

Univariate binary interface variables allow information to flow in only one direction. Interface variables with a larger number of values can capture more complex network dependencies that exhibit bidirectional information flow, multidimensional interface variables or noise correlations^38^. In particular, the lower performance in reconstructing the *E. coli* gene regulatory network compared to the in-silico benchmark may be due to noise correlations of expression levels caused by sample preparation, array fabrication, and array processing^39,40^. An extension to interface variables with four or more values seems feasible and promising, since the necessary conditions for corresponding functional modularizations are already derived in the Supp. Methods.

In general, exact probabilistic inference is intractable in large biological networks. We provide a hypothesis-driven statistical method that efficiently tests for selected functional modularizations and does not require complete information about the entire network. With recent advances in high-throughput single-cell technology^41^, multi-electrode array technology^42,43^, two-photon microscopy^44,45^ and genetically encoded voltage indicators^46,47^, our mathematical framework can be applied to a wide range of datasets to facilitate the analysis of complex biological systems.

## Methods

### Moments indicate functional modules

We show that moments of **s** uniquely determine whether the observable states form a particular modularization or not (see Supp. Methods). We consider raw mixed moments defined as an expectation of the corresponding monomials in **s**, e.g., *E*[*s*_1_*s*_2_].

The method is based on the following property of single functional modules. Let *P*_*n*_ for *n* = 1, 2, … and *Q*_*m*_ for *m* = 1, 2, … denote infinite sequences of all monomials in the observable components inside and outside of a given functional module, respectively. If the 2 × 2 matrix *M*_*ij*_ = *E*[*P*_*i*_*Q*_*j*_] for *i*, *j* = 1, 2 is invertible, then a necessary and sufficient condition for the existence of a binary interface variable *y* is

4 $$E[{P}_{n}{Q}_{m}]=\mathop{\sum}_{i,j=1,2}E[{P}_{n}{Q}_{j}]{\left({M}^{-1}\right)}_{ji}E[{P}_{i}{Q}_{m}]$$

for all *n*, *m* (see Lemma 1, Lemma 2 and Theorem 6 in the Supp. Methods).

Intuitively, the expectation *E*[*P*_*n*_*Q*_*m*_] is equal to the scalar product **p**_*n*_ ⋅ **q**_*m*_ of the two vectors **p**_*n*_ and **q**_*m*_ defined analogous to Eq. (1). The vector components are unknown because the interface variable *y* is unknown. However, **p**_*n*_ can be expressed in the skewed coordinate system with axes perpendicular to **q**_1_ and **q**_2_ and, likewise, **q**_*m*_ can be expressed in the skewed coordinate system with axes perpendicular to **p**_1_ and **p**_2_ (Fig. 4a). Evaluation of these coordinates by means of scalar products, transformation of the resulting vectors into the original orthogonal coordinate system by means of the matrix **M** and calculation of the scalar product results in Eq. (4).

A necessary and sufficient condition for the existence of a nested modularization is that this condition holds for each functional module in the modularization. Although a precise identification of modularizations requires an infinite number of moments, these conditions can be tested for finitely many moments to potentially falsify modularizations. Moreover, if the conditions for flat modularizations with moments as used in Eq. (3) hold, then there is always a flat modularization with these moments.

### A statistical test for modularizations

Let *N*^(t)^ denote the total number of i.i.d. samples of **s** and let ${\hat{{{{{{{{\bf{s}}}}}}}}}}^{(n)}$ for *n* ∈ {1, …, *N*^(t)^} denote the *n*-th sample. We assume *N*^(t)^ is even and the samples are normalized to zero mean. In this case, the test can be further simplified to the condition that certain ratios of moments of **s** have identical values if they are finite.

Let ${d}^{({{{{{{{{\mathcal{X}}}}}}}}}_{0})}$ denote the total number of moment ratios used for the test and let the components of ${{{{{{{\bf{b}}}}}}}}=({b}_{1},\ldots ,{b}_{{d}^{({{{{{{{{\mathcal{X}}}}}}}}}_{0})}})$ denote these ratios (see Eqs. (2) and (3)). For a given modularization ${{{{{{{\mathcal{M}}}}}}}}$, we introduce ${d}^{({{{{{{{{\mathcal{X}}}}}}}}})}$ index sets ${X}_{c}^{({{{{{{{\mathcal{M}}}}}}}})}$ for $c\in \{1,\ldots ,{d}^{({{{{{{{\mathcal{X}}}}}}}})}\}$ such that all components of **b** indexed by a set have the same value. The specific choice of moments, their ratios used for the test and the definition of the index sets ${X}_{c}^{({{{{{{{\mathcal{M}}}}}}}})}$ depend on the type of modularization, i.e., single functional module, flat or nested modularization, and is described in the sections below (see Supp. Methods for details).

For each sample moment ratio ${\hat{b}}_{v}$ for $v\in \{1,\ldots ,{d}^{({{{{{{{{\mathcal{X}}}}}}}}}_{0})}\}$ we define a vector ${\hat{{{{{{{{\bf{b}}}}}}}}}}^{(v)}\in {{\mathbb{R}}}^{2}$ containing the numerator and the denominator of ${\hat{b}}_{v}$. We estimate the moment ratio *b*_*v*_ by

5 $${\hat{b}}_{v}=\left\{\begin{array}{ll}0,\quad &\left\vert {\varrho }^{(v)}\right\vert \, < \, {\theta }_{\hat{\mu }}\\ \frac{{\hat{b}}_{1}^{(v)}}{{\hat{b}}_{2}^{(v)}},\quad &\,{{\mbox{otherwise}}}\,\end{array}\right.$$

6 $${\varrho }^{(v)}=\sqrt{\frac{{N}^{({{{{{{{\rm{t}}}}}}}})}}{2\hat{\Sigma }_{22}^{\prime (v,v)}}}{\hat{b}}_{2}^{(v)},$$

where a cutoff ${\theta }_{\hat{\mu }}=5$ ensures finite expectations of ${\hat{b}}_{v}$ for joint normal ${\hat{{{{{{{{\bf{b}}}}}}}}}}^{\left(v\right)}$.

The simplest possible moments for testing nested modularizations are ${b}_{1}^{\left(v\right)}=E[{s}_{k} \, {s}_{l}\, {s}_{d}]$ and ${b}_{2}^{\left(v\right)}=E[{s}_{k}\, {s}_{d}]E[{s}_{l} \, {s}_{d}]$ for *k* ≠ *l*, which are estimated by the sample moments

7 $${\hat{b}}_{i}^{\left(v\right)}=\frac{2}{{N}^{({{{{{{{\rm{t}}}}}}}})}}\mathop{\sum }_{n=1}^{{N}^{({{{{{{{\rm{t}}}}}}}})}/2}{\hat{\Phi }}_{ni}^{\left(v\right)}$$

8 $${\hat{\Phi }}_{n1}^{\left(v(k,l)\right)}={\hat{s}}_{k}^{(2n-1)}{\hat{s}}_{l}^{(2n-1)}{\hat{s}}_{d}^{(2n-1)}$$

9 $${\hat{\Phi }}_{n2}^{\left(v(k,d)\right)}={\hat{s}}_{k}^{(n)}{\hat{s}}_{d}^{(n)}{\hat{s}}_{l}^{(n+{N}^{({{{{{{{\rm{t}}}}}}}})}/2)}{\hat{s}}_{d}^{(n+{N}^{({{{{{{{\rm{t}}}}}}}})}/2)}$$

for *i* ∈ {1, 2}, *k* ∈ {1, …, *l* − 1}, *l* ∈ {2, …, *d* − 1} and vectorization

10 $$v(k,l)=\frac{(d-3)(d-2)}{2}-\frac{(d-2-k)(d-1-k)}{2}+l-1.$$

The corresponding sample covariance matrices are

11 $${\hat{\Sigma}}^{\prime (v,\omega )}_{ij}=\frac{1}{{N}^{({{{{{\rm{t}}}}}})}/2-1} \mathop{\sum }_{n=1}^{{N}^{({{{{{\rm{t}}}}}})}/2}\left({\hat{\Phi }}_{ni}^{\left(v\right)}-{\hat{b}}_{i}^{(v)}\right)\left({\hat{\Phi }}_{nj}^{\left(\omega \right)}-{\hat{b}}_{j}^{(\omega )}\right)$$

for *i*, *j* ∈ {1, 2} and $v,\omega \in \{1,\ldots ,{d}^{({{{{{{{{\mathcal{X}}}}}}}}}_{0})}\}$, where *ω*(*k*, *l*) = *v*(*k*, *l*). The covariance matrix of the sample moment ratios $\hat{{{{{{{{\bf{b}}}}}}}}}$ can be approximated by

12 $${\hat{\Sigma }}_{v\omega }=\left\{\begin{array}{ll}\infty , \hfill & \left(v=\omega \right)\,{{\mbox{and}}}\,\left(\left\vert {\varrho }^{(v)}\right\vert \, < \, {\theta }_{\hat{\mu }}\right) \hfill \\ 0, \hfill &\left(v\, \ne \, \omega \right)\,{{\mbox{and}}}\,\left(\left(\left\vert {\varrho }^{(v)}\right\vert \, < \, {\theta }_{\hat{\mu }}\right)\,{{\mbox{or}}}\,\left(\left\vert {\varrho }^{(\omega )}\right\vert \, < \, {\theta }_{\hat{\mu }}\right)\right)\hfill\\ \frac{2\sqrt{{\lambda }_{v}{\lambda }_{\omega }}}{{N}^{({{{{{{{\rm{t}}}}}}}})}{\hat{b}}_{2}^{(v)}{\hat{b}}_{2}^{(\omega )}}\left({\hat{\Sigma }}^{\prime (v,\omega )}_{11}-\frac{{\hat{b}}_{1}^{(\omega )}}{{\hat{b}}_{2}^{(\omega )}} {\hat{\Sigma }}^{\prime (v,\omega )}_{12}-\right. &\\ \left.\frac{{\hat{b}}_{1}^{(v)}}{{\hat{b}}_{2}^{(v)}} {\hat{\Sigma }}^{\prime (v,\omega )}_{21} + \frac{{\hat{b}}_{1}^{(v)}{\hat{b}}_{1}^{(\omega )}}{{\hat{b}}_{2}^{(v)}{\hat{b}}_{2}^{(\omega )}} {\hat{\Sigma }}^{\prime (v,\omega )}_{22} \right),\quad &\, {{{{{{\rm{otherwise}}}}}}}\hfill\,\end{array}\right.$$

for $$v,\omega \in \{1,\ldots ,{d}^{({{{{{{{{\mathcal{X}}}}}}}}}_{0})}\}$$.

The simplest possible moments for testing single functional modules (in gene regulatory networks) are ${b}_{1}^{\left(v\right)}=E[{s}_{k}\,{s}_{l}]$ and ${b}_{2}^{\left(v\right)}=E[{s}_{k}\, {s}_{d}]$ for *k* ≠ *l* estimated by

13 $${\hat{\Phi }}_{n1}^{\left(v(k,l)\right)}={\hat{s}}_{k}^{(n)}{\hat{s}}_{l}^{(n)}$$

14 $${\hat{\Phi }}_{n2}^{\left(v(k,l)\right)}={\hat{s}}_{k}^{(n)}{\hat{s}}_{d}^{(n)}.$$

We show that the statistic

15 $${{{{{{{{\mathcal{T}}}}}}}}}_{{{{{{{{\mathcal{M}}}}}}}}}(\hat{{{{{{{{\bf{b}}}}}}}}},\hat{{{{{{{{\bf{\Sigma }}}}}}}}})=\frac{1}{2}\mathop{\sum }_{k=1}^{{d}^{({{{{{{{{\mathcal{X}}}}}}}}}_{0})}}\frac{{\left({\hat{b}}_{k}\right)}^{2}}{{\hat{\Sigma }}_{kk}}-\frac{1}{2}{\sum }_{c=1}^{{d}^{({{{{{{{\mathcal{X}}}}}}}})}}\frac{{\left({\sum }_{l\in {X}_{c}^{({{{{{{{\mathcal{M}}}}}}}})}}{\hat{b}}_{l}{\left({\hat{\Sigma }}_{ll}\right)}^{-1}\right)}^{2}}{{\sum }_{l\in {X}_{c}^{({{{{{{{\mathcal{M}}}}}}}})}}{\left({\hat{\Sigma }}_{ll}\right)}^{-1}}$$

can be used to test for modularization ${{{{{{{\mathcal{M}}}}}}}}$ even if some moment ratios don’t exist because of zero denominators. The test is asymptotically correct for *λ*_*v*_ = 1. However, we consolidate numerically that the test also applies to finitely many samples if the moment estimates are approximately joint normal, ${N}^{({{{{{{{\rm{t}}}}}}}})}\hat{{{{{{{{\bf{\Sigma }}}}}}}}}\, \approx \, {N}^{({{{{{{{\rm{t}}}}}}}})}E[\hat{{{{{{{{\bf{\Sigma }}}}}}}}}]$ and

16 $${\lambda }_{v}={\xi }_{0}\left(1+\mathop{\sum }_{n=1}^{5}{\xi }_{n}{\left(\frac{{\varrho }^{(v)}}{6}\right)}^{-2n}\right),$$

where (*ξ*_0_, …, *ξ*_5_) = (1.367, 2.047, 4.735, −1.923, −1.231, 2.790).

For the most general test, we introduce a scaling factor ${\lambda }^{(\max )}$, which corrects for correlated components of $\hat{{{{{{{{\bf{b}}}}}}}}}$, and constrain the nominal significance level *α*^(*)^ to be larger than a minimal nominal significance level ${\alpha }^{(\min )}$, which corrects for potential zero correlations between *s*_ref_ and other observable components.

Let ${{{{{{{{\mathcal{M}}}}}}}}}_{i}$ denote the *i*-th of ${d}^{({{{{{{{\mathcal{H}}}}}}}})}$ modularizations tested on the same samples $\hat{{{{{{{{\bf{s}}}}}}}}}$. If the observable states form the modularization ${{{{{{{{\mathcal{M}}}}}}}}}_{i}$, the probability of sampling ${{{{{{{{\mathcal{T}}}}}}}}}_{{{{{{{{{\mathcal{M}}}}}}}}}_{i}}(\hat{{{{{{{{\bf{b}}}}}}}}},{\lambda }^{(\max )}\hat{{{{{{{{\bf{\Sigma }}}}}}}}})$ at least as extreme as observed is less than *α*^(*)^ if

17 $${p}_{i}^{(\Gamma )}\,{{\mbox{-value}}}\,\le \frac{{\alpha }^{(\Gamma )}}{{d}^{({{{{{{{\mathcal{H}}}}}}}})}}$$

18 $${\alpha }^{(\Gamma )}=\frac{{\alpha }^{(* )}-{\alpha }^{(\min )}}{1-{\alpha }^{(\min )}},$$

where ${p}_{i}^{(\Gamma )}\,{{\mbox{-value}}}\,$ denotes the probability of sampling ${{{{{{{{\mathcal{T}}}}}}}}}_{{{{{{{{{\mathcal{M}}}}}}}}}_{i}}(\hat{{{{{{{{\bf{b}}}}}}}}},{\lambda }^{(\max )}\hat{{{{{{{{\bf{\Sigma }}}}}}}}})$ at least as extreme as observed when distributed according to the one-sided right-tail gamma distribution $\Gamma ({\zeta }_{{{{{{{{{\mathcal{M}}}}}}}}}_{i}},1)$ for shape parameter ${\zeta }_{{{{{{{{\mathcal{M}}}}}}}}_{i}}=\frac{1}{2}\left({d}^{({{{{{{{{\mathcal{X}}}}}}}}}_{0})}-{d}^{({{{{{{{\mathcal{X}}}}}}}})}\right)$ and scale parameter 1. For ${\zeta }_{{{{{{{{\mathcal{M}}}}}}}}_{i}}=0$, ${{{{{{{{\mathcal{T}}}}}}}}}_{{{{{{{{\mathcal{M}}}}}}}}_{i}}=0$. ${\zeta }_{{{{{{{{\mathcal{M}}}}}}}}_{i}}$ corresponds to the degrees of freedom.

For potentially correlated components of the sample moment ratios $\hat{{{{{{{{\bf{b}}}}}}}}}$, we set ${\lambda }^{(\max )}$ such that the statistical test remains conservative. The smallest possible ${\lambda }^{(\max )}$ with this property is the largest eigenvalue of a submatrix of $\hat{{{{{{{{\bf{\Sigma }}}}}}}}}$ normalized to unit diagonal, i.e.,

19 $$\grave{{{{{\mathbf{\Sigma}}}}}}\hat{{{{{{{{\bf{\Sigma }}}}}}}}}\grave{{{{{\mathbf{\Sigma}}}}}},$$

where the matrix $\grave{{{{{\mathbf{\Sigma}}}}}}$ has the same size as $\hat{{{{{{{{\bf{\Sigma }}}}}}}}}$, is diagonal and ${\grave{\Sigma }}_{vv}={\hat{\Sigma }}_{vv}^{-1/2}$ if *v* is element of an index set of size larger than one and ${\grave{\Sigma }}_{vv}=0$, otherwise. A more power full test is derived in the Supp. Methods (see Section 2.4). The minimal nominal significance level

20 $${\alpha }^{(\min )}=1-\,{{\mbox{erf}}}\,{\left(\frac{{\theta }_{\hat{\mu }}-1}{\sqrt{2}}\right)}^{{d}^{({{{{{{{{\mathcal{X}}}}}}}}}_{0})}}\,{{\mbox{erf}}}\,{\left(\frac{6}{\sqrt{2}}\right)}^{{d}^{({{{{{{{{\mathcal{X}}}}}}}}}_{0})}},$$

where erf denotes the error function.

If all moment rations are finite, the test is asymptotically consistent for flat modularizations, i.e., its power for any incorrect modularization converges asymptotically to one. If in addition all moment ratios are uncorrelated, the test statistic is asymptotically minimal sufficient, i.e., it most efficiently captures all information about a modularization contained in the sample moment ratios. More details and the derivative of the statistical test can be found in the Supp. Methods. The performance of the test for detecting deviations from ideal functional modules, such as non-binary interface variables and additional dependencies, and for the case where moment ratios do not exist is shown in the Supp. Methods (see chapter 2.6).

Finally, if both conditions in the introduction are met, the inference for modularizations with binary and continuous interface variables is equivalent. Given that *s*_ref_ is considered as a single module, a modularization with continuous interface variables exists if and only if a corresponding modularization with binary interface variables and the same correlations as used for the test exist.

### Inference in gene regulatory networks

We participated in the transcriptional network inference challenge from DREAM5 that compares 35 methods for inference of gene regulatory networks: 29 submitted by participants and additional 6 off-the-shelf methods classified into six categories: Regression, Mutual information, Correlation, Bayesian networks, Meta (combinations of several different approaches), and Other (methods not belonging to any of the previous categories). The design of the challenge, detailed methods and results are reported elsewhere^16^.

We evaluate the network reconstruction from gene expression microarray datasets for *E. coli* and an in-silico benchmark using the area under the precision-recall (PR) curve, the area under the receiver operating characteristic (ROC) curve, and an overall score defined as the mean of the (log-transformed) network-specific *p* values (obtained by simulating a null distribution for 25000 random networks),

21 $${{{\mbox{score}}}}_{{{{{{{{\rm{ROC}}}}}}}}}=\frac{1}{2}\mathop{\sum }_{i=1}^{2}-{\log }_{10}\, {p}_{{{{{{{{\rm{ROC}}}}}}}},i}$$

22 $${{{\mbox{score}}}}_{{{{{{{{\rm{PR}}}}}}}}}=\frac{1}{2}\mathop{\sum }_{i=1}^{2}-{\log }_{10}\, {p}_{{{{{{{{\rm{PR}}}}}}}},i}$$

23 $$\,{{\mbox{score}}}=\frac{{{{\mbox{score}}}}_{{{{{{{{\rm{ROC}}}}}}}}}+{{{\mbox{score}}}}_{{{{{{{{\rm{PR}}}}}}}}}}{2}.$$

We omit the third dataset of the challenge for *S. cerevisiae* due to technical reasons, i.e., the size of the network is too large for the algorithm and computer hardware in use.

The *Escherichia coli* dataset consists of 4511 genes (334 TF) and a gold standard of 3766 TF-TG interactions (94% of 4012 total), according to which 89% of all indirect TF-TG interactions are removable. The in-silico datasets consist of 1643 genes (195 TF) and a gold standard of 1923 TF-TG interactions (94% of 2066 total), according to which 72% of all indirect TF-TG interactions are removable.

Given a sorted list of regulatory interaction *p* values in ascending order, we apply the test to any four-node subnetwork consisting of two TFs and two TGs. We call a certain number of regulatory interactions with the lowest ranks in the list the set of most likely interactions. We call the TF with the most likely interaction in a four-node subnetwork the putative interface variable. We call a subnetwork sufficiently connected if at least three of the four TF–TG interactions, including both with the putative interface variable, are in the set of the most likely interactions. If a subnetwork is sufficiently connected, then each *p* value of interactions with the other TF (not the putative interface variable) is changed by

24 $$p\,{{\mbox{-value}}}=\max (p{{\mbox{-value}}},c\cdot {p}^{{{{{{{{\rm{(test)}}}}}}}}}\,{{\mbox{-value}}}),$$

where *p*^(test)^-value denotes the *p *value of the test. For a scaling factor *c* = 1, the test is conservative for the null hypothesis (no direct interaction), which corresponds to the hypothesis ((no dependency) or (functional module)). We heuristically set *c* to the smallest *p* value of any interaction in the set of most likely interactions, ensuring that missing evidence against a functional module is not weighted more heavily than the evidence for interactions that determine whether the test is performed at all. The size of the set of most likely interactions is determined by a holdout set consisting of every 8th sample.

To combine tests for the same interaction in different subnetworks, we again take the maximum of the individual *p* values, which corresponds to a combined conservative test for the logic or operation of the individual hypotheses. If the gene regulatory network is i) nested such that each TG can only by reached from any TF via a single interface variable, ii) all TF–TG interactions are essential, i.e., the removal of a single TF-TG interaction results in additional independencies in **s**, and iii) indirect TF–TG interactions are less correlated than direct TF–TG interactions, then the network reconstruction is asymptotically correct in the sense that the most likely inferred interactions are all true TF–TG interactions.

More precisely, we test for the modularization ${{{{{{{\mathcal{M}}}}}}}}=\{S\}$ consisting of the single functional module *S* = {1, 2} (Fig. 3a) resulting in a single set ${X}^{({{{{{{{\mathcal{M}}}}}}}})}=\{1,2\}$ that indexes the only two components of the moment ratio vector *b*_*k*_ = *E*[*s*_*k*_*s*_3_]/*E*[*s*_*k*_*s*_ref_] for *k* ∈ {1, 2}. To avoid corrections for correlated components of the moment ratio vector, we estimate both *b*_*k*_ using disjoint sets of samples. Both datasets consist of *N*^(t)^ = 804 samples (microarrays). Furthermore, we use the uncorrected asymptotic version of the test, where *λ*_*v*_ = 1, and a small cutoff ${\theta }_{\hat{\mu }} \, < \, 1$.

To apply the method to a ranked list of *N* regulatory interactions, we artificially assign p values *rank*/*N*. For the sorted list of Pearson correlation coefficients, this procedure results in roughly the same AUPR, with even a slight performance improvement of 0.29%.

### Inference in pyramidal neurons

We simulated the detailed compartmental model of a CA1 pyramidal neuron developed by ref. ^24^ in the simulation environment NEURON. The model includes various active and passive membrane mechanisms, such as sodium and potassium currents, A-type potassium currents, m-type potassium currents, hyperpolarization-activated h-current, voltage-dependent calcium currents, and Ca^2+^-dependent potassium currents. The densities and distributions of these currents are based on published data. We are interested in subthreshold synaptic integration and block all spike-generating currents at the soma.

Synaptic inputs consist of an NMDA and an AMPA-type conductance with a ratio of their peak values of 2.5. Each of the 60 terminal branches contains ten synapses, with equal distances between adjacent synapses or branch ends. Each synapse is stimulated by a Poisson process at a constant rate of 32 Hz. The dendritic spike rate is ~28 Hz, which is in the range of values observed experimentally in neocortical pyramidal neurons from freely behaving rats^22^.

The datasets consist of the membrane potentials at the soma and the centers of the 26 most proximal terminal branches of the apical dendrites. Samples are recorded for 20 or 60 min at 50 ms time intervals, ensuring that their normalized autocovariance is less than 0.05. *N*^(t)^ is either 24,000 or 72,000.

The moment ratio vector $\hat{{{{{{{{\bf{b}}}}}}}}}$ and the index sets ${X}^{({{{{{{{\mathcal{M}}}}}}}})}$ are derived from the corresponding elements above the diagonal of $\hat{{{{{{{{\bf{B}}}}}}}}}$ (see Fig. 4f and Supp. Methods). To test for a purely linear functional module *S*, we define an additional set ${X}^{({{{{{{{\mathcal{M}}}}}}}})}$ that indexes all elements above the diagonal of the square submatrix of $\hat{{{{{{{{\bf{B}}}}}}}}}$ that is indexed by *S*. For each module or branch originating from the trunk, the largest *p* value is calculated over all combinations of modules and branches containing that module and consisting of 11 observable components to ensure identical degrees of freedoms.

The resulting flat modularization, which consists of the nonlinear functional modules *S*_4_ and *S*_7_ and a complementary linear somatic module (Fig. 6f), cannot be rejected at an overall significance level of 0.01. In contrast, the flat modularization consisting of the functional modules *S*_2_ and *S*_7_ and a complementary linear somatic module can be rejected at an overall significance level of 0.01.

### Inference in neural networks

The neural network consists of *d* = 5 populations, each with 10 neurons. For every 200 ms time interval, the firing rates of all neurons in the *i*-th population are chosen according to a binary random variable *x*_*i*_ ∈ {1, 2} for *i* ∈ {1, …, *d*} such that they are set to 5 Hz if *x*_*i*_ = 1 or 25 Hz, otherwise. The total number of spikes in the *i*-th population and the *n*-th time interval defines the observable component ${\hat{s}}_{i}^{(n)}$ (normalized to zero mean). *N*^(t)^ = 4500.

We implement the modularization shown in Fig. 5a, which consists of ${d}^{({{{{{{{\mathcal{M}}}}}}}})}=2$ functional modules, where *s*_ref_ refers to *s*_5_. The binary random vector **x** and the binary interface variables *y*_*c*_ ∈ {1, 2} for *c* ∈ {1, 2} are distributed according to

25 $$\,{{\mbox{P}}}\,({x}_{4}=2)=0.5$$

26 $$\,{{\mbox{P}}}\,({x}_{3}=2| {x}_{4})=\left\{\begin{array}{ll}0.85,\quad &{x}_{4}=2 \hfill \\ 0.15,\quad &{{\mbox{otherwise}}}\,,\end{array}\right.$$

27 $$\,{{\mbox{P}}}\,({x}_{2}=2| {y}_{1})=\left\{\begin{array}{ll}0.85,\quad &{y}_{1}=2\hfill \\ 0.15,\quad &{{\mbox{otherwise}}}\,,\end{array}\right.$$

28 $$\,{{\mbox{P}}}\,({x}_{1}=2| {y}_{2})=\left\{\begin{array}{ll}0.85,\quad &{y}_{2}=2\hfill \\ 0.15,\quad &{{\mbox{otherwise}}}\,,\end{array}\right.$$

29 $$\,{{\mbox{P}}}\,({y}_{1}=2| {x}_{3},{x}_{4})=\left\{\begin{array}{ll}1,\quad &{x}_{3}={x}_{4}=2\\ 0,\quad &{{\mbox{otherwise}}}\,,\hfill \end{array}\right.$$

30 $$\,{{\mbox{P}}}\,({y}_{2}=2| {x}_{2},{y}_{1})=\left\{\begin{array}{ll}0.85,\quad &{y}_{1}={x}_{2}=2 \hfill \\ 1,\quad &{y}_{1} \, \ne \, {x}_{2} \hfill \\ 0,\quad &{{\mbox{otherwise}}} \,, \hfill \end{array}\right.$$

31 $$\,{{\mbox{P}}}\,({x}_{5}=2| {x}_{1},{y}_{2})=\left\{\begin{array}{ll}1,\quad &{x}_{1}={y}_{2}=2 \hfill \\ 0,\quad &{{\mbox{otherwise}}}\,.\hfill \end{array}\right.$$

The rate functions for *y*_1_ and *x*_5_ are shown in the bottom left panel and the rate function for *y*_2_ is shown in the bottom right panel of Fig. 1b. An optimal linear model predicting *x*_5_ from the other components of **x** has a coefficient of determination of *R*^2^ = 0.79.

The moment ratio vector $\hat{{{{{{{{\bf{b}}}}}}}}}$ and the index sets ${X}^{({{{{{{{\mathcal{M}}}}}}}})}$ are derived from the corresponding elements above the diagonal of $\hat{{{{{{{{\bf{B}}}}}}}}}$ (see Fig. 4h and Supp. Methods). To test for an arbitrary linear modularization ${{{{{{{{\mathcal{M}}}}}}}}}_{{{{{{{{\rm{L}}}}}}}}}$, we define an additional set ${X}^{({{{{{{{{\mathcal{M}}}}}}}}}_{{{{{{{{\rm{L}}}}}}}}})}$ that indexes all elements above the diagonal of $\hat{{{{{{{{\bf{B}}}}}}}}}$.

**Fig. 7:**
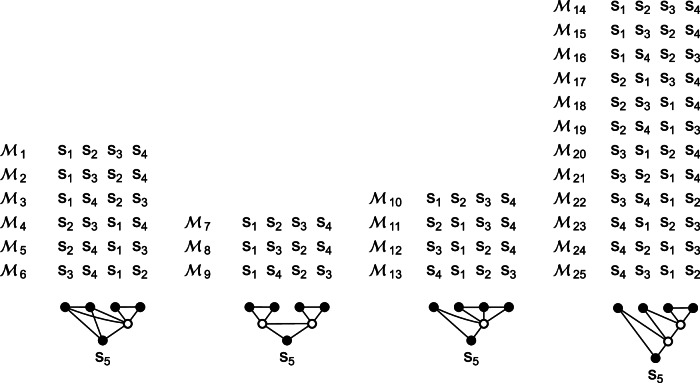
All possible modularizations of a network consisting of five components. All 25 modularizations ${{{{{{{{\mathcal{M}}}}}}}}}_{1}$ to ${{{{{{{{\mathcal{M}}}}}}}}}_{25}$ of an undirected graphical model consisting of five components *s*_1_ to *s*_5_, such that each functional model has at least two components.

All components of $\hat{{{{{{{{\bf{s}}}}}}}}}$ are correlated with Pearson correlation coefficients greater than 0.41 (Fig. 5c). To generate a linear modularization with the covariance matrix ${\hat{{{{{{{{\bf{\Sigma }}}}}}}}}}^{{{{{{{{\rm{(s)}}}}}}}}}$ of $\hat{{{{{{{{\bf{s}}}}}}}}}$, we apply the linear transformation **L** obtained from the Cholesky decomposition ${\hat{{{{{{{{\bf{\Sigma }}}}}}}}}}^{{{{{{{{\rm{(s)}}}}}}}}}={{{{{{{\bf{L}}}}}}}}{{{{{{{{\bf{L}}}}}}}}}^{T}$ to the time-shuffled observable states (shuffled independently for each population). All tests are repeated 10^4^ times on independent datasets. All 25 modularizations are shown in Fig. 7.

### Reporting summary

Further information on research design is available in the Nature Portfolio Reporting Summary linked to this article.

### Supplementary information

Supplementary Information

Reporting Summary

## Data Availability

The datasets analyzed during the current study are available at Zenodo^48^.

## Code availability

An implementation of the statistical test in MATLAB (R2015a) is available at Zenodo^48^.
